# Serum levels of mature microRNAs in *DICER1*-mutated pleuropulmonary blastoma

**DOI:** 10.1038/oncsis.2014.1

**Published:** 2014-02-10

**Authors:** M J Murray, S Bailey, K L Raby, H K Saini, L de Kock, G A A Burke, W D Foulkes, A J Enright, N Coleman, M Tischkowitz

**Affiliations:** 1 Department of Paediatric Haematology and Oncology, Addenbrooke's Hospital, Cambridge, UK; 2 Department of Pathology, University of Cambridge, Cambridge, UK; 3 European Molecular Biology Laboratory-European Bioinformatics Institute (EMBL-EBI), Cambridge, UK; 4 Program in Cancer Genetics, Department of Oncology and Human Genetics, McGill University, Montreal, QC, Canada; 5 Department of Histopathology, Addenbrooke's Hospital, Cambridge, UK; 6 Department of Medical Genetics, University of Cambridge, Cambridge, UK

**Keywords:** *DICER1*, microRNA, pleuropulmonary blastoma, serum

## Abstract

*DICER1* is a critical gene in the biogenesis of mature microRNAs, short non-coding RNAs that derive from either -3p or -5p precursor microRNA strands. Germline mutations of *DICER1* are associated with a range of human malignancies, including pleuropulmonary blastoma (PPB). Additional somatic ‘hotspot' mutations in the microRNA processing ribonuclease IIIb (RNase IIIb) domain of *DICER1* are reported in cancer, and which affect microRNA biogenesis, resulting in a -3p mature microRNA strand bias. Here, in a germline (exon11 c.1806_1810insATTGA) *DICER1*-mutated PPB, we first confirmed the presence of an additional somatic RNase IIIb hotspot mutation (exon25 c.5425G>A [p.G1809R]) by conventional sequencing. Second, we investigated serum levels of mature microRNAs at the time of PPB diagnosis, and compared the findings with serum results from a comprehensive range of pediatric cancer patients and controls (*n*=52). We identified a panel of 45 microRNAs that were present at elevated levels in the serum at the time of PPB diagnosis, with a significant majority noted be derived from the -3p strand (*P*=0.013). In addition, we identified a subset of 10 serum microRNAs (namely miR-125a-3p, miR-125b-2-3p, miR-380-5p, miR-125b-1-3p, *let-7f*-2-3p, *let-7a*-3p, *let-7b*-3p, miR-708-3p, miR-138-1-3p and miR-532-3p) that were most abundant in the PPB case. Serum levels of two representative microRNAs, miR-125a-3p and miR-125b-2-3p, were not elevated in *DICER1* germline-mutated relatives. In the PPB case, serum levels of miR-125a-3p and miR-125b-2-3p increased before chemotherapy, and then showed an early reduction following treatment. These microRNAs may offer future utility as serum biomarkers for screening patients with known germline *DICER1* mutations for early detection of PPB, and for potential disease-monitoring in cases with confirmed PPB.

## Introduction

*DICER1* is a member of the ribonuclease III (RNase III) family and is critically involved in the biogenesis of microRNAs, short non-protein-coding RNAs that negatively regulate protein-coding gene expression, as well as other functions that include chromatin regulation and alternative splicing.^1^ DICER1 is a 1922-amino-acid protein that contains, among other important domains, two RNase III domains (IIIa and IIIb) toward the C terminus, responsible for the processing of microRNA precursor molecules. *DICER1* mutations may be germline or somatic in nature. Germline mutations occur throughout the gene, although the functional consequences are variable.^2^ Although the protein from the affected allele is predicted to be truncated or not produced at all, owing to nonsense-mediated decay, some DICER1 protein may still be produced.^2^ In contrast, somatic ‘hotspot' mutations generally occur in the RNase IIIb domain.^1, 3^ In humans, heterozygous germline mutations of *DICER1* are associated with several childhood cancers, such as the rare lung tumor pleuropulmonary blastoma (PPB),^4^ Wilms tumor, cystic nephroma and other conditions such as multinodular goiter.^5^ Recently, biallelic *DICER1* mutations have been demonstrated to account for a small proportion of Wilms tumor.^1^

PPBs are composed of malignant mesenchymal cells, often associated with benign epithelium.^4^ The majority of children with PPB are found to have germline *DICER1* mutations, although penetrance of these mutations is low.^6^ Relatively little is known about the potential mechanisms through which mutations in the *DICER1* gene may lead to tumor development. However, most data are consistent with a standard two-hit model of tumorigenesis, where the first germline mutation is followed at a later stage by a second somatic mutation, almost exclusively in the RNase IIIb domain, in certain cell types (e.g. those of the lung or thyroid) that subsequently manifest as organ-specific disease (e.g. PPB or goiter). Immunohistochemical analysis for DICER1 protein expression in various tumors from patients known to harbor germline *DICER1* mutations has yielded inconsistent results for both biological and technical reasons.^4, 5^ In PPB cases, for example, expression from the wild-type *DICER1* allele has been shown to be lost in tumor epithelium, but is retained in the malignant mesenchymal cells,^4^ and differences in the sensitivity and specificity of individual DICER1 antibodies remain to be fully characterized.^5^

The RNase III domains of the DICER1 protein process precursor stem-loop microRNAs (pre-microRNAs) in the cytoplasm to produce single-stranded, mature microRNAs (18–23 nucleotides (nt) in length). Mature microRNAs arising from the 5′ end of the stem-loop precursor are termed -5p microRNAs, and those from the 3′ end as -3p microRNAs. These are incorporated into the RNA-induced silencing complex, where they regulate gene expression through binding to messenger RNA targets.^7^ *DICER1* mutations of the RNase IIIb domain are associated with altered microRNA processing, in particular a strand bias toward the production of -3p rather than -5p microRNAs, owing to the loss of -5p strand cleavage of the pre-microRNA.^3^

Importantly, microRNA profiles have been shown to classify human cancers.^8^ For example, we have shown that microRNA profiles universally segregate malignant germ cell tumors (GCTs) from non-malignant controls^9^ and that the same microRNAs are detectable at high levels in the serum of patients at the time of malignant GCT diagnosis,^10, 11, 12^ findings recently replicated by others.^13, 14^ These diagnostic serum microRNA profiles are sensitive enough to detect malignant GCT disease at tumor volumes as low as 4 cm^3^.^11^

**Figure 1:**
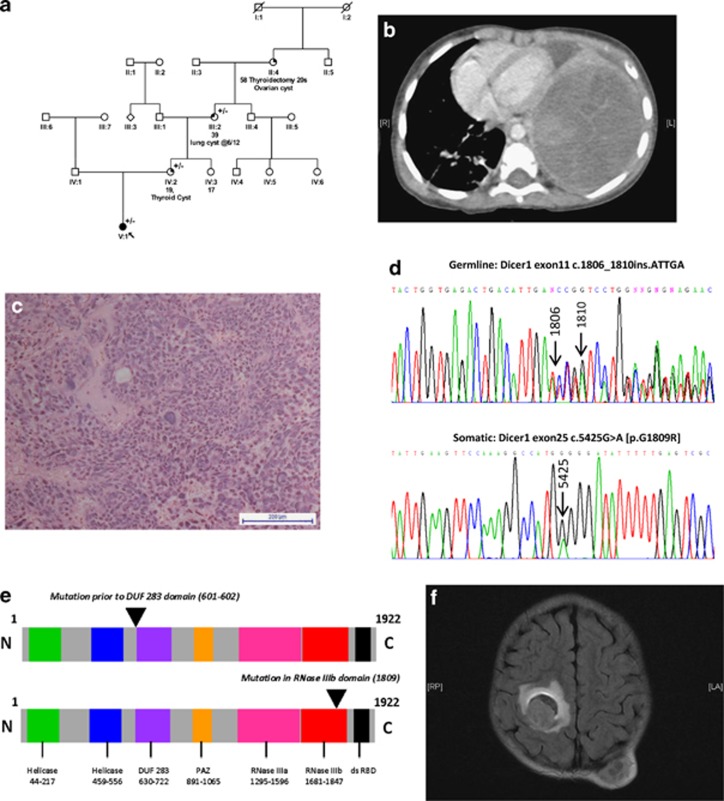
Clinicopathological data for the *DICER1*-mutated PPB case. (**a**) Family tree showing the history consistent with a germline *DICER1* mutation on the maternal side (+/−=heterozygous for the germline mutation). (**b**) Chest CT scan at presentation revealed a large left-sided chest mass, causing displacement of the heart and compression of the left lung. (**c**) Representative hematoxylin and eosin stain showing the Type III PPB, taken at × 20 magnification using a Nikon Eclipse TS100 microscope and a Nikon DS-Fi1 camera (Nikon UK Ltd, Kingston upon Thames, UK). (**d**) Electrophoretogram showing the germline heterozygous c.1806_1810insATTGA *DICER1* mutation (upper panel) and the somatic c.5425 G>A [p.G1809R] *DICER1* RNase IIIb domain mutation (lower panel). Tumor DNA was extracted and *DICER1* RNase IIIa and RNase IIIb domains screened for ‘hotspot' mutations, as described.^1^ Polymerase chain reaction (PCR) amplification of the regions of interest was performed as described^24^ and sequencing undertaken by the McGill University and Genome Quebec Innovation Center (MUGQIC) using conventional Sanger sequencing methods. (**e**) Schematic of the DICER1 protein showing site of the germline-truncating mutation (upper image) and somatic RNase IIIb domain mutation (lower image). (**f**) Magnetic resonance imaging brain scan showing the right-sided cerebral metastasis and the left occipital bony metastatic deposit.

Here, we studied a 2-year-old girl at the time of PPB diagnosis, who was found to harbor a germline *DICER1* mutation. She initially presented with a 1-week history of cough and fever. Family history was suggestive of a germline *DICER1* mutation (Figure 1a). Chest computed tomography (CT) scan confirmed a large left-sided chest mass (Figure 1b). Lytic metastatic deposits were noted in the thoracic spine and ribs. Biopsy confirmed a ‘Type III' PPB (http://www.ppbregistry.org/) (Figure 1c). Mutation analysis of DNA from lymphocytes identified a heterozygous germline *DICER1* insertion mutation, c.1806_1810insATTGA (Figure 1d, top panel), resulting in a frameshift and therefore presumed to be deleterious (Figure 1e, top panel). The same mutation was subsequently identified in the patient's mother and maternal grandmother, in keeping with the family history. Staging investigations confirmed further metastatic bony deposits, including in the occipital region. Chemotherapy was commenced according to the PPB Registry treatment recommendations (http://www.ppbregistry.org/) and 12 courses were delivered over 9 months with objective radiological response. However, reassessment at the end of treatment, including magnetic resonance imaging of the head, revealed a new right-sided cerebral metastasis (Figure 1f). She received palliative whole brain radiotherapy, but presented acutely 10 days later with left-sided weakness. A computed tomography head scan confirmed an increase in size of the right-sided cerebral metastasis. She received palliative support and died 1 year after initial presentation.

For this study, we firstly screened tumor tissue for the presence of an additional somatic mutation of the RNase IIIa and RNase IIIb domains. We then quantified global levels of mature microRNAs in the serum at the time of diagnosis of PPB, comparing findings with serum results from pediatric cancer patients and controls (*n*=52). We compared results with those from the proband's mother and maternal grandmother who both carried the same germline *DICER1* mutation and demonstrated that PPB-specific serum microRNAs showed an early response to treatment. We have identified microRNAs that may offer future utility as serum biomarkers for screening patients with known germline *DICER1* mutations for early detection of PPB, and for potential disease monitoring in cases with confirmed PPB.

## Results and discussion

Analysis of the *DICER1* RNase IIIa and RNase IIIb domains in tumor tissue confirmed a somatic ‘hotspot' mutation within exon 25, resulting in a c.5425G>A [p.G1809R] mutation affecting the RNase IIIb domain (Figure 1d, bottom panel). This mutation has been observed previously in a PPB case (Foulkes *et al.*, unpublished) and is predicted to result in replacement of a glycine with arginine at position 1809 of the 1922-amino-acid DICER1 protein. This mutation is predicted to reduce RNase IIIb activity, likely to result in abnormal processing of microRNA precursor molecules in affected tissues (Figure 1e, bottom panel). This finding is consistent with the demonstration that somatic RNase IIIb hotspot mutations of *DICER1* occur in association with germline mutations, particularly loss-of-function mutations.^3^ Mutant DICER1 proteins resulting from somatic mutations generally have reduced RNase IIIb activity but retain RNase IIIa activity,^15^ and are associated with defective microRNA processing and -3p strand bias.^3^ It is believed that such mutations in epithelial lung tissue result in the subsequent development of PPB in pulmonary mesenchymal cells,^4^ although the precise mechanisms remain to be characterized.

**Figure 2:**
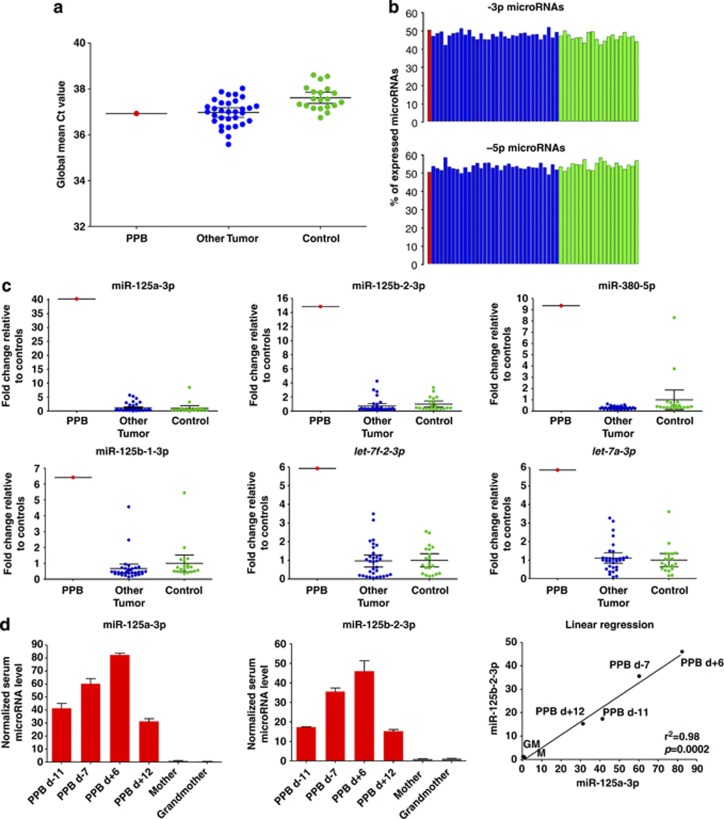
Serum mature microRNAs in the *DICER1*-mutated PPB case. (**a**) The graph shows global mean Ct values for serum mature microRNAs (*n*=568) in the PPB case at the time of diagnosis (red; left) compared with mean values for the reference tumors of childhood (without a family history suggestive of germline *DICER1* mutations) (*n*=32; blue; center) and age- and gender-matched control samples from subjects without malignancy (*n*=20; green; right). Serum samples were processed^10^ before RNA extraction (miRNeasy Mini Kit; Qiagen, Crawley, UK), as described.^25^ The RNA eluate was used for universal reverse transcription using miRCURY-LNA microRNA reagents (Exiqon, Vedbaek, Denmark). The universal cDNA was diluted 50 × , before quantitative reverse transcription polymerase chain reaction (qRT–PCR) for 741 human microRNAs using the Exiqon microRNA Ready-to-Use PCR Human-Panels I and II in a LightCycler 480 Real-Time-PCR System (Roche, Welwyn Garden City, UK), including spike-in controls for quality control. MicroRNAs with Ct values ⩽37 were considered expressed. Samples had no evidence of significant hemolysis, by visual inspection and analysis of miR-451 levels, as described.^26^ Data were normalized using the global mean method, that is, the average Ct value of microRNAs expressed in at least one of the 53 samples analyzed (*n*=568), as described.^27^ MicroRNA levels were then quantified using the delta Ct method. MicroRNAs that had a ⩾2.0 fold change in expression in the PPB case compared with the mean expression value from a) the control group and b) the ‘other tumor' group were considered overexpressed. (**b**) The graph shows the expression of -3p (top panel) and -5p (lower panel) microRNAs as a percentage of the total number of expressed microRNAs. The PPB case at the time of diagnosis (red column) is compared with percentages for individual ‘other tumor' samples (blue) and controls (green). Only expressed microRNAs with both -3p and -5p probes present on the Exiqon qRT–PCR platform (*n*=286) were considered for this analysis. (**c**) Levels of the six top-ranking most abundant microRNAs (from Table 1) for the *DICER1*-mutated PPB sample (red) when compared with other childhood tumor samples (blue) and controls (green). (**d**) The graphs show separate validation of the normalized serum levels of miR-125a-3p (left) and miR-125b-2-3p (center) and linear regression of these two microRNAs (right) in four longitudinal serum samples from the PPB case (red) and from serum from relatives with the same germline *DICER1* mutation (mother and maternal grandmother; gray). For this work, the non-human spike-in microRNA cel-miR-39-3p was used for quality control, before levels were normalized using three independently transcribed housekeeping microRNAs that were identified in the global study as having the most stable expression in the serum across the whole cohort (miR-191-5p, miR-30b-5p and miR-30c-5p). This independent qRT–PCR was performed using Taqman reagents and assays (Applied Biosystems, Warrington, UK) as per the manufacturer's instructions using a Realplex Mastercycler epgradient S (Eppendorf, Stevenage, UK). Results were then referenced to serum microRNA levels from the mother. Time-points listed are relative to the start of chemotherapy treatment, assigned as d 0. d, Day; M, mother; GM, maternal grandmother.

Following global microRNA profiling of the diagnostic serum sample from the *DICER1*-mutated PPB case, the normalized Ct data showed that there were similar levels of serum mature microRNAs (*n*=568) to other tumors of childhood and to control samples (Figure 2a). Of the 568 expressed microRNAs, a total of 286 had both -3p and -5p probes present on the Exiqon platform, for which there was a very similar expression level of both strands in all 53 samples analyzed (Figure 2b). This finding suggested that a germline mutation *per se* was not sufficient to cause a general reduction in mature microRNA levels in the bloodstream. In body tissues only affected by a single germline *DICER1* mutation, the second *DICER1* allele appears to permit enough functional DICER1 protein to be produced to maintain mature microRNA levels, reflected by similar abundances in the serum.

These findings are consistent with the notion that the major contributions to overall serum levels of mature microRNAs within the body in patients with PPB are from tissues that only harbor a single germline mutation, with no evidence of microRNA strand bias.

We next hypothesized that the large PPB tumor (Figure 1b) would release specific microRNAs into the bloodstream, which would be detectable as an altered serum profile compared with control serum samples and with diagnostic serum samples from the time of diagnosis of other childhood tumors. In total, we identified a list of 45 mature microRNAs that were overexpressed, that is, were at levels ⩾2.0-fold higher in the serum at *DICER1*-mutated PPB diagnosis, compared with both the cohort of ‘other tumors' of childhood and the control group (Supplementary Table S1). Given the direct evidence of a somatic *DICER1* RNase IIIb mutation in this PPB case, and the known association with defective microRNA processing,^3^ we next looked for evidence of a -3p strand bias in the overexpressed microRNAs in the serum from the *DICER1*-mutated PPB case. Of the original list of 45 overexpressed microRNAs, 14 had no specific -3p or -5p strand information and a further microRNA (miR-1237-3p) had no corresponding -5p probe on the test platform (Supplementary Table S1). The remaining 30 microRNAs had both -3p and -5p probes represented on the platform, of which 21 were derived from the -3p strand and only nine from the -5p strand (*P*=0.013; exact binomial test) (Supplementary Table S1).

Table 1 lists the 10 microRNAs (from the original list of 45) that were the most abundant in the PPB case compared with all the other 52 samples analyzed. Of these, nine were from the -3p strand and only one from the -5p strand (*P*=0.0098). These data indicate the robust presence of a -3p strand bias among overexpressed PPB-associated serum microRNAs. Levels of these 10 microRNAs were between 3.4- and 40-fold higher in the serum of the PPB patient when compared with mean serum levels in the control group (Table 1). The most substantial increase was for miR-125a-3p, with levels over 40- and 33-fold higher than the control group and the ‘other tumor' group, respectively. We next interrogated whether the 10 serum microRNAs that were elevated at PPB diagnosis have also been reported in other pulmonary malignancies. Of note, 8 of the 10 microRNAs showed altered expression in non-small-cell lung cancer (NSCLC) in adults, either in tumor tissue or patient serum at diagnosis (Table 1).^16, 17, 18, 19, 20, 21^ The six top-ranking microRNAs from our subset of 10 (miR-125a-3p, miR-125b-2-3p, miR-380-5p, miR-125b-1-3p, *let-7f*-2-3p and *let-7a*-3p) are represented graphically in Figure 2c.

To determine whether serum levels of representative microRNAs were PPB-specific or indicative only of changes due to germline *DICER1* mutations, we compared the findings for the two highest ranking differentially expressed microRNAs (miR-125a-3p and miR-125b-2-3p) with levels from two family members (mother and maternal grandmother) known to harbor the same germline *DICER1* mutation that was detected in the patient (Figure 2d). Although serum levels of miR-125a-3p and miR-125b-2-3p were similarly low in the mother and grandmother, levels were 41- and 17-fold higher, respectively, in the PPB case at diagnosis (day (d) −11 before treatment commenced), compared with levels in the mother, confirming their specificity for PPB (Figure 2d). These changes were similar to the fold changes seen for these microRNAs in the PPB case compared with the pediatric control samples (Table 1).

We next analyzed serum levels of miR-125a-3p and miR-125b-2-3p at the four time-points from which serum was available in the PPB case, representing two time-points before the delivery of chemotherapy (i.e. during diagnostic work-up), and two following. We observed that the levels of these two representative microRNAs increased during diagnostic work-up and briefly ‘flared' on commencement of chemotherapy (Figure 2d) (as we have observed for serum microRNAs in other tumor types (data not shown)), before rapidly falling at d +12 to levels below those seen at baseline assessment (d −11). Finally, using linear regression, we showed that levels of miR-125a-3p and miR-125b-2-3p were highly correlated (*P*=0.0002) (Figure 2d, right panel), suggesting that changes in the levels of these two independently transcribed microRNAs (Table 1) were disease-associated.

Of the lung malignancy-associated microRNAs, high tissue expression levels of miR-708 are strongly associated with an increased risk of death from NSCLC after adjustments for all clinically significant risk factors, including patient age, gender and tumor stage.^16^ MicroRNAs including miR-125a^17^ and members of the *let-7* family^18, 19^ have been shown to be significantly downregulated in NSCLC tumor samples, with low levels of expression associated with poor clinical outcomes. The observation that levels of these microRNAs may be downregulated in lung malignancy (compared with normal lung tissue), but detected at elevated levels in the serum (as shown here for PPB), is consistent with other findings. For example, miR-206, a muscle-specific microRNA, is downregulated in rhabdomyosarcoma samples (compared with normal skeletal muscle) and associated with poor overall survival,^22^ but found at elevated levels in the serum when compared with normal controls.^23^ Derangement of normal cellular function, allowing passive leakage of such microRNAs from tumor cells into the bloodstream, may be one explanation for these observations. Alternatively, active and selective microRNA release mechanisms from tumor cells could account for the described findings, which may in turn promote an environment suitable for tumor cell growth and metastasis. To this end, miR-125b has been shown to be highly expressed in the serum of patients with NSCLC, with levels correlating with tumor stage,^21^ whereas miR-138 has been associated with the development of cisplatin resistance in NSCLC.^20^ The PPB-associated serum microRNAs identified here are therefore likely to reflect both an origin from malignant lung tissue and a contribution from biallelic *DICER1* mutations, resulting in microRNA processing changes and a -3p strand bias. It will be important in the future to study serum microRNA profiles in other non-pulmonary *DICER1*-mutated tumors, to quantify such relative contributions more precisely.

We acknowledge the potential limitations of our study. We did not interrogate for microRNA profiles in the PPB tissue, owing to a lack of normal lung parenchyma samples for suitable comparison. First, however, as microRNAs that are detected at altered levels in the serum^10, 11, 12^ are often dysregulated in malignant tissues,^9^ serum profiling may be used directly to identify candidate biomarkers. Second, it will be important in the future to confirm these serum findings in a larger cohort of PPB cases, as we have done for malignant GCTs,^10, 11, 12^ as, to the best of our knowledge, we are not aware that any such PPB serum sample set currently exists. Finally, it is possible that a very small minority of tumor cases in our comparison group may have been from patients carrying undetected germline *DICER1* mutations, as we only performed gene sequencing in the PPB case. However, it should be noted that the family history obtained in the non-PPB tumor cases was not suggestive of such mutations and that germline PPB mutations are rare, even in individuals with *DICER1-*associated non-PPB tumors such as Wilms tumor and rhabdomyosarcoma.

If confirmed in future studies, we propose that the panel of PPB-associated microRNAs identified here may represent useful screening biomarkers for PPB in patients known to have germline *DICER1* mutations and those with a suggestive family history. If so, this approach may reduce or obviate the need for regular surveillance imaging, with the associated risks of radiation exposure (for computed tomography scans) and/or general anesthesia and sedation (required to perform magnetic resonance imaging scans in young children). In confirmed PPB cases, it will be important to identify whether levels of these serum microRNAs accurately reflect disease activity and may therefore offer potential for disease monitoring. Further functional studies are also required to identify fully how *DICER1* mutations result in the development of PPB.

## Figures and Tables

**Table 1:** The most abundant serum mature microRNAs in *DICER1*-mutated PPB

*MicroRNA and miRBase accession number*	*5′–3′ nucleotide sequence*	*Chromosomal location(s)*	*PPB fold change*	*‘Other tumor' fold change*	*Implication in NSCLC*	*Publication*
miR-125a-3p (MIMAT0004602)	ACAGGUGAGGUUCUUGGGAGCC	19q13.41	40.28	1.19	Tissue levels correlate with stage and metastases	Jiang *et al.*^17^
miR-125b-2-3p (MIMAT0004603)	UCACAAGUCAGGCUCUUGGGAC	21q21.1	14.84	0.71	High serum levels correlate with poor prognosis	Yuxia *et al.*^21^
miR-380-5p (MIMAT0000734)	UGGUUGACCAUAGAACAUGCGC	14q32.31	9.26	0.30	N/A	N/A
miR-125b-1-3p (MIMAT0004592)	ACGGGUUAGGCUCUUGGGAGCU	11q24.1	6.41	0.66	High serum levels correlate with poor prognosis	Yuxia *et al.*^21^
*let-7f*-2-3p (MIMAT0004487)	CUAUACAGUCUACUGUCUUUCC	Xp11.22	5.92	0.96	Tissue levels correlate with reduced survival	Takamizawa *et al.*^19^
*let-7a*-3p (MIMAT0004481)	CUAUACAAUCUACUGUCUUUC	9q22.32; 22q13.31	5.87	1.11	Tissue levels correlate with shortened survival	Takamizawa *et al.*^19^
*let-7b-*3p (MIMAT0004482)	CUAUACAACCUACUGCCUUCCC	22q13.31	4.57	1.23	Tissue levels correlate with worse survival	Jusofovic *et al.*^18^
miR-708-3p (MIMAT0004927)	CAACUAGACUGUGAGCUUCUAG	11q14.1	4.15	0.78	Tissue levels correlate with increased risk of death	Jang *et al.*^16^
miR-138-1-3p (MIMAT0004607)	GCUACUUCACAACACCAGGGCC	3p21.32	3.95	0.56	Role in cisplatin resistance	Wang *et al.*^20^
miR-532-3p (MIMAT0004780)	CCUCCCACACCCAAGGCUUGCA	Xp11.23	3.43	1.58	N/A	N/A

Abbreviations: N/A, not available; NSCLC, non-small-cell lung cancer; PPB, pleuropulmonary blastoma.

The table lists the 10 microRNAs that were most abundant in the serum in the *DICER1*-mutated PPB case at the time of diagnosis compared with the control group and the other childhood tumors (‘other tumors') group. It also provides information on sequence, chromosomal location and fold change in the PPB case and ‘other tumor' samples referenced to the normal control samples. Further information is provided for the microRNAs that have been implicated in adult lung malignancy.

## References

[bib1] Wu M Sabbaghian N Xu B Addidou-Kalucki S Bernard C Zou D Biallelic DICER1 mutations occur in Wilms tumours J Pathol 2013 230 154 164 23620094 10.1002/path.4196

[bib2] Rio Frio T Bahubeshi A Kanellopoulou C Hamel N Niedziela M Sabbaghian N DICER1 mutations in familial multinodular goiter with and without ovarian Sertoli–Leydig cell tumors JAMA 2011 305 68 77 21205968 10.1001/jama.2010.1910 PMC3406486

[bib3] Anglesio MS Wang Y Yang W Senz J Wan A Heravi-Moussavi A Cancer-associated somatic DICER1 hotspot mutations cause defective miRNA processing and reverse-strand expression bias to predominantly mature 3p strands through loss of 5p strand cleavage J Pathol 2013 229 400 409 23132766 10.1002/path.4135

[bib4] Hill DA Ivanovich J Priest JR Gurnett CA Dehner LP Desruisseau D DICER1 mutations in familial pleuropulmonary blastoma Science 2009 325 965 19556464 10.1126/science.1174334 PMC3098036

[bib5] Foulkes WD Bahubeshi A Hamel N Pasini B Asioli S Baynam G Extending the phenotypes associated with DICER1 mutations Hum Mutat 2011 32 1381 1384 21882293 10.1002/humu.21600

[bib6] Slade I Bacchelli C Davies H Murray A Abbaszadeh F Hanks S DICER1 syndrome: clarifying the diagnosis, clinical features and management implications of a pleiotropic tumour predisposition syndrome J Med Genet 2011 48 273 278 21266384 10.1136/jmg.2010.083790

[bib7] Gregory RI Chendrimada TP Cooch N Shiekhattar R Human RISC couples microRNA biogenesis and posttranscriptional gene silencing Cell 2005 123 631 640 16271387 10.1016/j.cell.2005.10.022

[bib8] Lu J Getz G Miska EA Alvarez-Saavedra E Lamb J Peck D MicroRNA expression profiles classify human cancers Nature 2005 435 834 838 15944708 10.1038/nature03702

[bib9] Palmer RD Murray MJ Saini HK van Dongen S Abreu-Goodger C Muralidhar B Malignant germ cell tumors display common microRNA profiles resulting in global changes in expression of messenger RNA targets Cancer Res 2010 70 2911 2923 20332240 10.1158/0008-5472.CAN-09-3301 PMC3000593

[bib10] Murray MJ Halsall DJ Hook CE Williams DM Nicholson JC Coleman N Identification of microRNAs From the miR-371∼373 and miR-302 clusters as potential serum biomarkers of malignant germ cell tumors Am J Clin Pathol 2011 135 119 125 21173133 10.1309/AJCPOE11KEYZCJHT

[bib11] Murray MJ Coleman N Testicular cancer: a new generation of biomarkers for malignant germ cell tumours Nat Rev Urol 2012 9 298 300 22549310 10.1038/nrurol.2012.86

[bib12] Gillis AJ Rijlaarsdam MA Eini R Dorssers LC Biermann K Murray MJ Targeted serum miRNA (TSmiR) test for diagnosis and follow-up of (testicular) germ cell cancer patients: a proof of principle Mol Oncol 2013 7 1083 1092 24012110 10.1016/j.molonc.2013.08.002 PMC5528443

[bib13] Belge G Dieckmann KP Spiekermann M Balks T Bullerdiek J Serum levels of microRNAs miR-371-3: a novel class of serum biomarkers for testicular germ cell tumors Eur Urol 2012 61 1068 1069 22386195 10.1016/j.eururo.2012.02.037

[bib14] Dieckmann KP Spiekermann M Balks T Flor I Loning T Bullerdiek J MicroRNAs miR-371-3 in serum as diagnostic tools in the management of testicular germ cell tumours Br J Cancer 2012 107 1754 1760 23059743 10.1038/bjc.2012.469 PMC3493876

[bib15] Heravi-Moussavi A Anglesio MS Cheng SW Senz J Yang W Prentice L Recurrent somatic DICER1 mutations in nonepithelial ovarian cancers N Engl J Med 2012 366 234 242 22187960 10.1056/NEJMoa1102903

[bib16] Jang JS Jeon HS Sun Z Aubry MC Tang H Park CH Increased miR-708 expression in NSCLC and its association with poor survival in lung adenocarcinoma from never smokers Clin Cancer Res 2012 18 3658 3667 22573352 10.1158/1078-0432.CCR-11-2857 PMC3616503

[bib17] Jiang L Huang Q Zhang S Zhang Q Chang J Qiu X Hsa-miR-125a-3p and hsa-miR-125a-5p are downregulated in non-small cell lung cancer and have inverse effects on invasion and migration of lung cancer cells BMC Cancer 2010 10 318 20569443 10.1186/1471-2407-10-318 PMC2903529

[bib18] Jusufovic E Rijavec M Keser D Korosec P Sodja E Iljazovic E Let-7b and miR-126 are down-regulated in tumor tissue and correlate with microvessel density and survival outcomes in non-small-cell lung cancer PLoS One 2012 7 e45577 23029111 10.1371/journal.pone.0045577 PMC3454421

[bib19] Takamizawa J Konishi H Yanagisawa K Tomida S Osada H Endoh H Reduced expression of the let-7 microRNAs in human lung cancers in association with shortened postoperative survival Cancer Res 2004 64 3753 3756 15172979 10.1158/0008-5472.CAN-04-0637

[bib20] Wang Q Zhong M Liu W Li J Huang J Zheng L Alterations of microRNAs in cisplatin-resistant human non-small cell lung cancer cells (A549/DDP) Exp Lung Res 2011 37 427 434 21787234 10.3109/01902148.2011.584263

[bib21] Yuxia M Zhennan T Wei Z Circulating miR-125b is a novel biomarker for screening non-small-cell lung cancer and predicts poor prognosis J Cancer Res Clin Oncol 2012 138 2045 2050 22806310 10.1007/s00432-012-1285-0 PMC11824208

[bib22] Missiaglia E Shepherd CJ Patel S Thway K Pierron G Pritchard-Jones K MicroRNA-206 expression levels correlate with clinical behaviour of rhabdomyosarcomas Br J Cancer 2010 102 1769 1777 20502458 10.1038/sj.bjc.6605684 PMC2883695

[bib23] Miyachi M Tsuchiya K Yoshida H Yagyu S Kikuchi K Misawa A Circulating muscle-specific microRNA, miR-206, as a potential diagnostic marker for rhabdomyosarcoma Biochem Biophys Res Commun 2010 400 89 93 20696132 10.1016/j.bbrc.2010.08.015

[bib24] Witkowski L Lalonde E Zhang J Albrecht S Hamel N Cavallone L Familial rhabdoid tumour ‘avant la lettre'—from pathology review to exome sequencing and back again J Pathol 2013 231 35 43 23775540 10.1002/path.4225

[bib25] Exiqon. Profiling of microRNA in serum/plasma and other biofluids. Available at http://www.exiqon.com/ls/Documents/Scientific/microRNA-serum-plasma-guidelines.pdf (accessed 1 October 2013).

[bib26] Kirschner MB Kao SC Edelman JJ Armstrong NJ Vallely MP van Zandwijk N Haemolysis during sample preparation alters microRNA content of plasma PLoS One 2011 6 e24145 21909417 10.1371/journal.pone.0024145 PMC3164711

[bib27] Mestdagh P Van Vlierberghe P De Weer A Muth D Westermann F Speleman F A novel and universal method for microRNA RT–qPCR data normalization Genome Biol 2009 10 R64 19531210 10.1186/gb-2009-10-6-r64 PMC2718498

