# Supplementary material for: Serum levels of mature microRNAs in DICER1-mutated pleuropulmonary blastoma
Source: Oncogenesis. 2014 Feb 10;3(2):e87–. doi: 10.1038/oncsis.2014.1 (PMC3940920; doi:10.1038/oncsis.2014.1)
Supplement: Supplementary Table 1 [file oncsis20141x1.doc]

| **Rank** | **microRNA** | **-3p & -5p probe** | **Highest in PPB** | **PPB fold change** | **‘Other Tumor’ fold change** | **Control fold change** | **Sequence**  **(Accession number)** | **Chromosomal location** |
| --- | --- | --- | --- | --- | --- | --- | --- | --- |
| 1 | hsa-miR-125a-3p | Y | Y | 40.28 | 1.19 | 1.0 | ACAGGUGAGGUUCUUGGGAGCC  (MIMAT0004602) | 19q13.41 |
| 2 | hsa-miR-125b-2-3p | Y | Y | 14.84 | 0.71 | 1.0 | UCACAAGUCAGGCUCUUGGGAC  (MIMAT0004603) | 21q21.1 |
| 3 | hsa-miR-380-5p | Y | Y | 9.26 | 0.30 | 1.0 | UGGUUGACCAUAGAACAUGCGC  (MIMAT0000734) | 14q32 |
| 4 | hsa-miR-99b-3p | Y | N | 7.24 | 1.35 | 1.0 | CAAGCUCGUGUCUGUGGGUCCG  (MIMAT0004678) | 19q13.41 |
| 5 | hsa-miR-25-5p | Y | N | 6.95 | 1.76 | 1.0 | AGGCGGAGACUUGGGCAAUUG  (MIMAT0004498) | 7q22.1 |
| 6 | hsa-miR-2113 | N/A | N | 6.92 | 1.69 | 1.0 | AUUUGUGCUUGGCUCUGUCAC  (MIMAT0009206) | 6q16.1 |
| 7 | hsa-miR-125b-1-3p | Y | Y | 6.41 | 0.66 | 1.0 | ACGGGUUAGGCUCUUGGGAGCU  (MIMAT0004592) | 11q24.1 |
| 8 | hsa-miR-196b-3p | Y | N | 6.09 | 2.86 | 1.0 | UCGACAGCACGACACUGCCUUC  (MIMAT0009201) | 7p15.2 |
| 9 | hsa-miR-661 | N/A | N | 5.96 | 1.12 | 1.0 | UGCCUGGGUCUCUGGCCUGCGCGU  (MIMAT0003324) | 8q24.3 |
| 10 | hsa-*let-7f*-2-3p | Y | Y | 5.92 | 0.96 | 1.0 | CUAUACAGUCUACUGUCUUUCC  (MIMAT0004487) | Xp11.22 |
| 11 | hsa-*let-7a*-3p | Y | Y | 5.87 | 1.11 | 1.0 | CUAUACAAUCUACUGUCUUUC  (MIMAT0004481) | 22q13.31 |
| 12 | hsa-miR-188-3p | Y | N | 5.60 | 1.66 | 1.0 | CUCCCACAUGCAGGGUUUGCA  (MIMAT0004613) | Xp11.23 |
| 13 | hsa-miR-130b-3p | Y | N | 5.46 | 2.67 | 1.0 | CAGUGCAAUGAUGAAAGGGCAU  (MIMAT0000691) | 22q11.31 |
| 14 | hsa-miR-1237-3p | N | N | 5.34 | 1.25 | 1.0 | UCCUUCUGCUCCGUCCCCCAG  (MIMAT0005592) | 11q13.1 |
| 15 | hsa-miR-654-3p | Y | N | 5.30 | 1.86 | 1.0 | UAUGUCUGCUGACCAUCACCUU  (MIMAT0004814) | 14q32.31 |
| 16 | hsa-miR-515-3p | Y | N | 4.81 | 1.74 | 1.0 | GAGUGCCUUCUUUUGGAGCGUU  (MIMAT0002827) | 19q13.42 |
| 17 | hsa-miR-10b-3p | Y | N | 4.63 | 1.10 | 1.0 | ACAGAUUCGAUUCUAGGGGAAU  (MIMAT0004556) | 2q31.1 |
| 18 | hsa-miR-520e | N/A | N | 4.62 | 0.88 | 1.0 | AAAGUGCUUCCUUUUUGAGGG  (MIMAT0002825) | 19q13.42 |
| 19 | hsa-*let-7b*-3p | Y | Y | 4.57 | 1.23 | 1.0 | CUAUACAACCUACUGCCUUCCC  (MIMAT0004482) | 22q13.31 |
| 20 | hsa-miR-18b-3p | Y | N | 4.49 | 0.60 | 1.0 | UGCCCUAAAUGCCCCUUCUGGC  (MIMAT0004751) | Xq26.2 |
| 21 | hsa-miR-1249 | N/A | N | 4.20 | 1.46 | 1.0 | ACGCCCUUCCCCCCCUUCUUCA  (MIMAT0005901) | 22q13.31 |
| 22 | hsa-miR-708-3p | Y | Y | 4.15 | 0.78 | 1.0 | CAACUAGACUGUGAGCUUCUAG  (MIMAT0004927) | 11q14.1 |
| 23 | hsa-miR-23a-5p | Y | N | 4.14 | 1.24 | 1.0 | GGGGUUCCUGGGGAUGGGAUUU  (MIMAT0004496) | 19p13.13 |
| 24 | hsa-miR-1972 | N/A | N | 4.12 | 1.88 | 1.0 | UCAGGCCAGGCACAGUGGCUCA  (MIMAT0009447) | 16p13.11 |
| 25 | hsa-miR-214-3p | Y | N | 4.11 | 1.04 | 1.0 | ACAGCAGGCACAGACAGGCAGU  (MIMAT0000271) | 1q24.3 |
| 26 | hsa-miR-103b | N/A | N | 4.08 | 0.94 | 1.0 | UCAUAGCCCUGUACAAUGCUGCU  (MIMAT0007402) | 5q34 |
| 27 | hsa-miR-1913 | N/A | N | 4.05 | 1.30 | 1.0 | UCUGCCCCCUCCGCUGCUGCCA  (MIMAT0007888) | 6q27 |
| 28 | hsa-miR-138-1-3p | Y | Y | 3.95 | 0.56 | 1.0 | GCUACUUCACAACACCAGGGCC  (MIMAT0004607) | 3p21.32 |
| 29 | hsa-miR-760 | N/A | N | 3.73 | 0.97 | 1.0 | CGGCUCUGGGUCUGUGGGGA  (MIMAT0004957) | 1p22.1 |
| 30 | hsa-miR-532-3p | Y | Y | 3.44 | 1.58 | 1.0 | CCUCCCACACCCAAGGCUUGCA (MIMAT0004780) | Xp11.23 |
| 31 | hsa-miR-572 | N/A | N | 3.32 | 1.08 | 1.0 | GUCCGCUCGGCGGUGGCCCA  (MIMAT0003237) | 4p15.33 |
| 32 | hsa-miR-512-3p | Y | N | 3.31 | 0.88 | 1.0 | AAGUGCUGUCAUAGCUGAGGUC  (MIMAT0002823) | 19q13.42 |
| 33 | hsa-miR-493-3p | Y | N | 3.31 | 1.53 | 1.0 | UGAAGGUCUACUGUGUGCCAGG  (MIMAT0003161) | 14q32.2 |
| 34 | hsa-miR-449b-3p | Y | N | 3.25 | 1.14 | 1.0 | CAGCCACAACUACCCUGCCACU  (MIMAT0009203) | 5q11.2 |
| 35 | hsa-miR-1537 | N/A | N | 3.24 | 1.58 | 1.0 | AAAACCGUCUAGUUACAGUUGU (MIMAT0007399) | 1q42.3 |
| 36 | hsa-miR-616-5p | Y | N | 2.99 | 1.09 | 1.0 | ACUCAAAACCCUUCAGUGACUU (MIMAT0003284) | 12q13.3 |
| 37 | hsa-miR-887 | N/A | N | 2.99 | 1.26 | 1.0 | GUGAACGGGCGCCAUCCCGAGG (MIMAT0004951) | 5p15.1 |
| 38 | hsa-miR-1911-5p | Y | N | 2.88 | 0.55 | 1.0 | UGAGUACCGCCAUGUCUGUUGGG  (MIMAT0007885) | Xq23 |
| 39 | hsa-miR-619 | N/A | N | 2.62 | 0.77 | 1.0 | GACCUGGACAUGUUUGUGCCCAGU  (MIMAT0003288) | 12q24.11 |
| 40 | hsa-miR-320b | N/A | N | 2.54 | 1.05 | 1.0 | AAAAGCUGGGUUGAGAGGGCAA  (MIMAT0005792) | 1p13.1 |
| 41 | hsa-miR-338-5p | Y | N | 2.40 | 0.95 | 1.0 | AACAAUAUCCUGGUGCUGAGUG  (MIMAT0004701) | 17q25.3 |
| 42 | hsa-miR-127-5p | Y | N | 2.36 | 0.90 | 1.0 | CUGAAGCUCAGAGGGCUCUGAU  (MIMAT0004604) | 14q32.2 |
| 43 | hsa-miR-629-5p | Y | N | 2.24 | 1.08 | 1.0 | UGGGUUUACGUUGGGAGAACU (MIMAT0004810) | 15q23 |
| 44 | hsa-miR-662 | N/A | N | 2.20 | 0.70 | 1.0 | UCCCACGUUGUGGCCCAGCAG  (MIMAT0003325) | 16p13.3 |
| 45 | hsa-miR-221-5p | Y | N | 2.08 | 0.78 | 1.0 | ACCUGGCAUACAAUGUAGAUUU  (MIMAT0004568) | Xp11.3 |

**Supplementary Table 1. *DICER1*-mutated PPB-associated serum mature microRNAs.** The Table lists the 45 over-expressed microRNAs that were present in the serum in the *DICER1*-mutated PPB case at the time of diagnosis compared with the control group and other childhood tumors (‘other tumors’) group. The Table provides information on whether individual microRNAs had both their corresponding -3p and -5p probes present on the Exiqon platform, their nucleotide sequence, chromosomal location and fold change in the PPB case and ‘other tumor’ samples referenced to the normal control samples. Key: Y=yes; N=no; N/A=not applicable.
